# Anticoagulation approach in morbid obesity: a comprehensive review on venous thromboembolism management

**DOI:** 10.3389/fphar.2024.1457280

**Published:** 2024-12-17

**Authors:** Victorita Sorodoc, Andreea Asaftei, Alexandr Ceasovschih, Catalina Lionte, Simina Crisan, Mihai Constantin, Lucia Indrei, Laurentiu Sorodoc

**Affiliations:** ^1^ 2nd Internal Medicine Department, Sf. Spiridon Clinical Emergency Hospital, Iasi, Romania; ^2^ Internal Medicine Department, Faculty of Medicine, Grigore T. Popa University of Medicine and Pharmacy, Iasi, Romania; ^3^ USTACC Department, Institute of Cardiovascular Diseases Timisoara, Timisoara, Romania; ^4^ Cardiology Department, Faculty of Medicine, Victor Babes University of Medicine and Pharmacy, Timisoara, Romania; ^5^ Radiology and Medical Imaging Department, Sf. Spiridon Clinical Emergency Hospital, Iasi, Romania

**Keywords:** anticoagulation, morbid obesity, venous thromboembolism, warfarin, unfractionated heparin, low-molecular-weight heparin, direct oral anticoagulant, bariatric

## Abstract

Obesity is a recognized risk factor for venous thromboembolism (VTE), associated with distinct challenges in managing anticoagulation therapy. There is still limited evidence regarding the impact of extreme body weight on the pharmacokinetics, pharmacodynamics, efficacy, and safety of various anticoagulant medications. To our knowledge, this is the first comprehensive review to address both prophylactic and therapeutic anticoagulant dosages specifically for managing VTE in patients with a body mass index (BMI) ≥40 kg/m^2^ or weight ≥120 kg. Our aim was to synthesize the findings of relevant studies alongside the latest recommendations on anticoagulation in this unique population. We gathered and analyzed data on all classes of anticoagulants available for VTE management, including vitamin K antagonists (VKAs), unfractionated heparin (UFH), low-molecular-weight heparin (LMWH), fondaparinux, and direct oral anticoagulants (DOACs), offering insights into their efficacy and safety profiles. Additionally, we explored special subpopulations of morbidly obese patients, such as those with cancer, renal dysfunction, or those undergoing bariatric surgery, recognizing the nuanced therapeutic challenges they present. The current evidence for anticoagulant therapy in morbidly obese patients with VTE is evidently insufficient, underscoring the need for a tailored approach and meticulous monitoring to achieve an optimal therapeutic balance.

## 1 Introduction

The prevalence of obesity has increased worldwide in the last decades, and it is widely recognized as a global epidemic (Boutari and Mantzoros, 2022). The most used classification for overweight and obesity is the one developed by the World Health Organization (WHO), which is based on the BMI (Aronne, 2002). BMI is calculated by dividing weight (in kilograms) by square height (in meters). A normal BMI is considered to be between 18.5 kg/m^2^ and 24.9 kg/m^2^, with overweight status falling between 25 kg/m^2^ and 29.9 kg/m^2^. Obesity is defined as a BMI ≥30 kg/m^2^, being divided in three classes: class 1 obesity–BMI between 30.0 kg/m^2^ and 34.9 kg/m^2^, class two obesity–BMI between 35.0 kg/m^2^ and 39.9 kg/m^2^, and class 3 obesity, also known as morbid obesity–BMI ≥40 kg/m^2^ (Aronne, 2002).

Epidemiologic studies have reported an association between high BMI and an extensive range of chronic diseases, such as cardiovascular disease, type 2 diabetes mellitus, non alcoholic fatty liver disease, sleep apnea, osteoarthritis, mental disorders, and even some types of cancer, leading to a decline in both quality of life and life expectancy (Blüher, 2019; Perez-Campos et al., 2020).

VTE includes both deep vein thrombosis (DVT) and pulmonary embolism (PE) (Ntinopoulou et al., 2022). It is associated with a significant risk of recurrence, chronic complications, such as post-thrombotic syndrome (PTS) and chronic thromboembolic pulmonary hypertension, and substantial mortality (Winter et al., 2017). Under physiological conditions, there exists an equilibrium between procoagulant and anticoagulant factors within the circulatory system, thus acting as a preventive mechanism against intravascular thrombus formation (Badireddy and Mudipalli, 2020). VTE results from the interaction of patient-specific risk factors and the particular clinical context in which the event occurs (Ntinopoulou et al., 2022). Virchow’s Triad succinctly categorizes the large number of VTE risk factors into three fundamental pathophysiological mechanisms that may precipitate thrombus formation: venous stasis, endothelial injury, and hypercoagulability (Kumar et al., 2010). In many cases, VTE is a preventable disease, and an early risk stratification of patients, particularly the identification of high-risk patients, may lead to the implementation of more effective prevention and therapeutic strategies (Ntinopoulou et al., 2022; Pastori et al., 2023). Even though there are cases of VTE occurring without any apparent reason, the majority of VTE cases exhibit one or more identifiable risk factors that either precipitate or contribute to the occurrence of VTE (Pastori et al., 2023).

Based on comprehensive observational studies, VTE risk factors have been classified into categories of weak, moderate and strong by the 2019 European Society of Cardiology (ESC) Guidelines for the diagnosis and management of acute PE developed in collaboration with the European Respiratory Society (ERS) (Konstantinides et al., 2019). Even though obesity is considered a minor risk factor for VTE, most patients present with additional VTE risk factors, considering the multiple comorbidities associated with obesity, as well as the reduced mobility of patients with morbid obesity (Amin et al., 2023; Pastori et al., 2023). Obesity is associated with an increased risk of VTE through several mechanisms, including a sedentary lifestyle, increased intra-abdominal pressure, diminished blood flow velocity within the lower extremities, as well as inflammatory and metabolic dysregulations that lead to a hypercoagulable state (Amin et al., 2023).

In terms of anticoagulant treatment recommendations for patients with obesity and VTE, uncertainties have persisted over the past decade regarding the effective and safe dosages of various anticoagulants. Due to alterations in pharmacokinetic parameters, anticoagulation medication may require dose adjustments. However, notable advancements have been made, particularly in patients with a BMI below 40 kg/m^2^, with concise guideline recommendations. Regarding patients with a BMI higher than 40 kg/m^2^, the optimal dosage has not been established for most anticoagulants.

The aim of this review is to summarize findings from relevant studies and recommendations on anticoagulation in patients with morbid obesity and VTE. We aim for this article to serve as a synthesis of available literature data, capable of guiding clinicians in adopting the most appropriate anticoagulation approach.

## 2 Obesity and VTE risk

Among the numerous metabolic abnormalities associated with obesity, the primary pathways predominantly responsible for obesity-induced venous thrombosis are represented by chronic inflammation and impaired fibrinolysis (Blokhin and Lentz, 2013).

**FIGURE 1:**
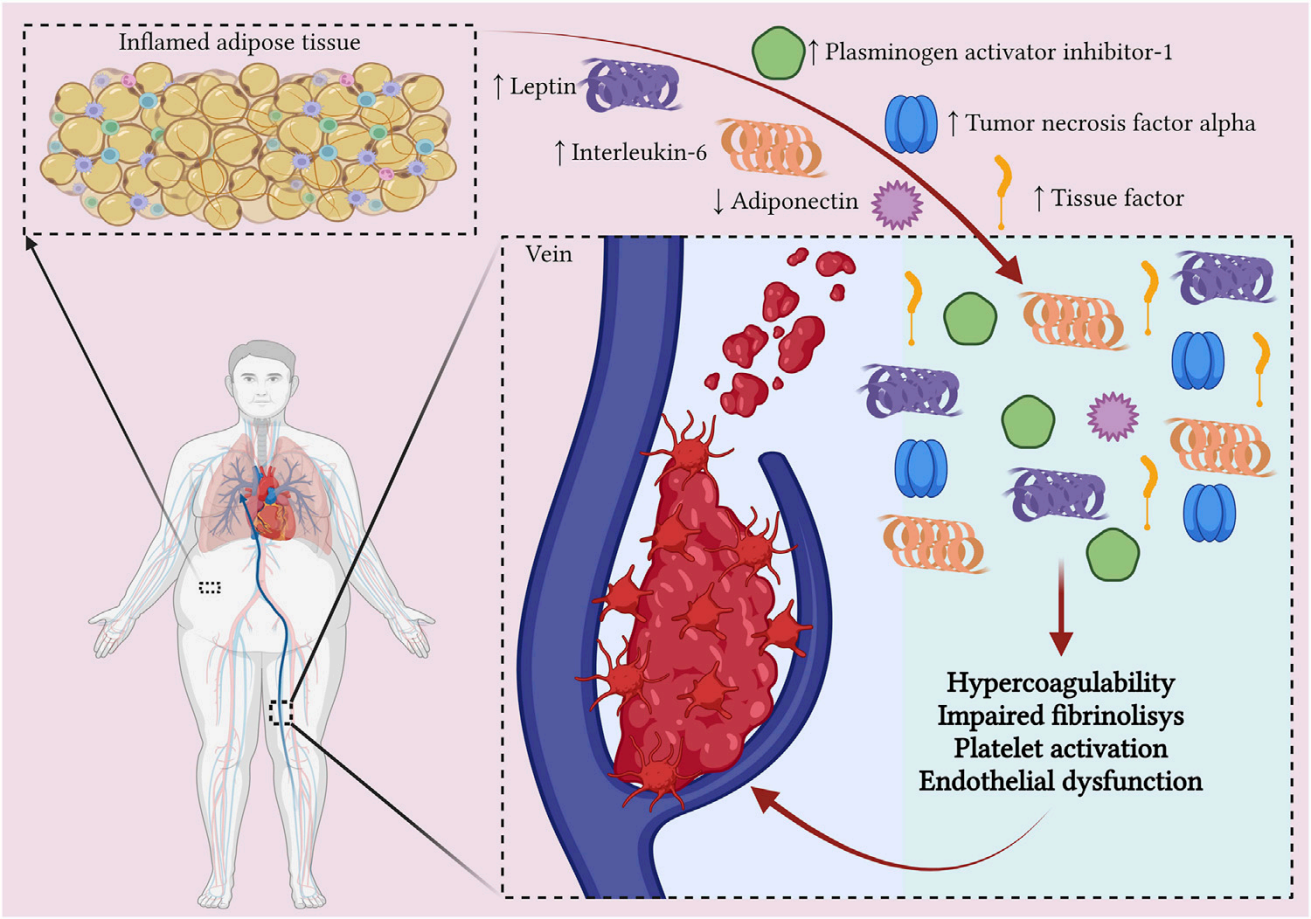
The relationship between obesity and venous thromboembolism. Adipocytes from obese individuals induce a low-grade inflammatory process by secreting pro-inflammatory cytokines. Additionally, the activated macrophages in adipose tissue, together with the pro-inflammatory cytokines, stimulate the expression of tissue factor, Factor VIII, and von Willebrand factor, promoting a pro-coagulable state. The altered adipose tissue also secretes elevated levels of plasminogen activator inhibitor-1, leading to impaired fibrinolysis. Moreover, obesity is associated with low levels of anti-inflammatory adipokines, such as adiponectin, which results in platelet activation (Created with BioRender.com).

Obesity also disrupts the regulation of several modulators of hemostatic balance, such as adipokines (Leal and Mafra, 2013; Ji and Guo, 2019) (Figure 1).

In recent years, studies have shown that obesity is linked to a low-grade inflammatory process, marked by elevated levels of pro-inflammatory cytokines such as interleukin-6 (IL-6), tumor necrosis factor alpha (TNF-alpha), and acute-phase proteins such as C-reactive protein (CRP) (Rodríguez-Hernández et al., 2013). These pro-inflammatory cytokines are secreted by adipocytes and lead to the recruitment of macrophages to adipose tissue (Rodríguez-Hernández et al., 2013). Additionally, transient hypoxia occurring in expanding adipose tissue further promotes macrophage recruitment (Blokhin and Lentz, 2013). The inflamed and altered adipose tissue environment can induce the polarization of macrophages towards a proinflammatory M1 phenotype as well as redirection of Th2 cells towards Th1 and Th17 types. This process results in the induction and maintenance of a systemic inflammatory response which also involves the liver and blood vessels (Vilahur et al., 2017). Moreover, the activated macrophages and pro-inflammatory cytokines can stimulate the expression of tissue factor (TF), which contributes to a pro-coagulable state (Rodríguez et al., 2015).

Obesity has also been associated with elevated levels of fibrinogen, factor VIII and von Willebrand factor, due to the action of pro-inflammatory cytokines on hepatocytes and endothelial cells (Kluin-Nelemans et al., 2012).

Plasminogen activator inhibitor-1 (PAI-1) plays a critical role in fibrinolysis by irreversibly inhibiting plasminogen activators, such as tissue plasminogen activator and uroplasminogen activator (Wang L et al., 2018). Elevated levels of PAI-1 were found in obese patients, with visceral adipose tissue having a greater capacity to produce PAI-1 than subcutaneous adipose tissue (Skurk and Hauner, 2004). Some studies associated elevated plasma levels of PAI-1 with an increased risk of future incident VTE in the obese population (Frischmuth T et al., 2022).

Adipokines are cell-signaling proteins produced by adipose tissue that play roles in controlling inflammation, immune responses, metabolism, cardiovascular function, and various other physiological processes (Clemente-Suárez et al., 2023). To date, hundreds of adipokines with different effects have been described. In obesity, there is an activation of pro-inflammatory adipokines, with the development of a low-grade inflammation, along with a suppression of anti-inflammatory adipokines (Kirichenko TV et al., 2022). Leptin is one of the best-known pro-inflammatory adipokines, exhibiting elevated circulating levels in the obese population. It has been associated with a higher risk of VTE by stimulating platelet activation, along with impairing fibrinolysis and thrombus resolution (Broni et al., 2023). Resistin is another pro-inflammatory adipokine found in higher levels in the obese population and associated with an increased risk of VTE due to its prothrombotic effects (Ding and Li, 2019). In contrast, adiponectin is an anti-inflammatory adipokine with antithrombotic effects. Decreased total adiponectin levels are found in the obese population, with multiple studies showing an increased risk of VTE as a result (Gariballa et al., 2019; Xiao et al., 2023).

## 3 VKAs use in morbidly obese patients with VTE

VKAs, classified into coumarin derivatives (e.g., warfarin, acenocoumarol) and indanedione derivatives (e.g., fluindione, phenidione), are widely used for preventing and managing thromboembolic disorders (Comets et al., 2012). They inhibit vitamin K epoxide reductase, reducing the synthesis of vitamin K-dependent coagulation factors (II, VII, IX, X) and the anticoagulant proteins C and S. Despite their efficacy, VKAs have some limitations, including delayed onset, a narrow therapeutic range, bleeding risks, and numerous drug and dietary interactions, requiring regular monitoring and dose adjustments (Caterina et al., 2013). The most frequently used vitamin K antagonist is warfarin, even though acenocoumarol, fenprocumon, or fluindione are still prescribed in some regions (Comets et al., 2012; Trautmann and Seitz, 2010). However, studies focused on the use of these less commonly prescribed VKAs in obesity are limited, as most of them have focused on warfarin.

### 3.1 Warfarin use in morbidly obese patients with VTE

Warfarin, the most used VKA, is one of the most commonly prescribed anticoagulants, with proven efficacy in preventing thromboembolic events in patients with atrial fibrillation or cardiac valve replacement, as well as in the prophylaxis and treatment of VTE (Tadros and Shakib, 2010).

International normalized ratio (INR) testing has a critical role in evaluating the balance between clinical efficacy and minimizing the risk of bleeding. The therapeutic range is typically between 2 and 3, with variations depending on individual clinical situations (McDowell et al., 2018). The response to warfarin is influenced by various factors, including medications, diet, and individual patient characteristics (McDowell et al., 2018). A higher BMI was found to be associated with a need for higher warfarin doses and with a longer time to obtain a therapeutic INR, due to higher volume of distribution and increased clearance (Kabagambe et al., 2013; Ogunsua et al., 2015).

The first study to evaluate INR stratified by BMI category in hospitalized patiens was made by Wallace et al. (2013). They conducted a retrospective study involving 211 patients who were newly initiated on warfarin therapy, categorizing them by BMI. They highlighted that obese and morbidly obese patients had a diminished initial response to warfarin compared to patients with a normal BMI. Furthermore, these patients were less likely to achieve a therapeutic INR before discharge within the same timeframe. They suggested that in obese and morbidly obese patients, 40%–50% higher initial warfarin doses may be needed (Wallace et al., 2013).

In 2014, Mueller et al. conducted a study in order to establish an association between BMI and the total weekly dose of warfarin (Mueller et al., 2014). They included 831 patients, with a BMI range between 13.4 and 63.1 kg/m^2^, who were taking warfarin and had an INR within the therapeutic range. They showed that for each 1-point increase in BMI, the weekly warfarin dose increased by 0.69 mg (Mueller et al., 2014). The need for a higher weekly dose of warfarin in morbidly obese patients was also demonstrated in a retrospective cohort study (RCS) conducted by Tellor et al. (2018). They also highlighted the importance of drug-drug interactions, noting that individuals concomitantly taking amiodarone required lower doses of warfarin in the same BMI class (Tellor et al., 2018). Alshammari et al. conducted a RCS, including 301 patients with a maintained therapeutic INR level, and who received a stable dose of warfarin over 3 months (Alshammari et al., 2020). They were categorized in 3 BMI groups (normal, overweight, and obese). It was found that obese patients had a significantly higher dose of warfarin (by around 20%) than those with a normal or overweight BMI (*p* = 0.013) (Alshammari et al., 2020).

In a more recent study, Soyombo et al. compared warfarin requirements across five different BMI categories, finding a statistically significant difference among the warfarin requirements to maintain a therapeutic INR across all BMI categories (*p* = 0.006) (Soyombo et al., 2021). What was interesting in their research is that included patients with a BMI ≥40 kg/m^2^ had a lower average warfarin dose when compared with obesity class I and II categories. This was due to a higher rate of nonsteroidal anti-inflammatory drugs (NSAIDs) and antiplatelet use in the morbidly obese patient group, underscoring the significance of concomitant medications and their potential impact on warfarin therapy and INR management (Soyombo et al., 2021).

**FIGURE 2:**
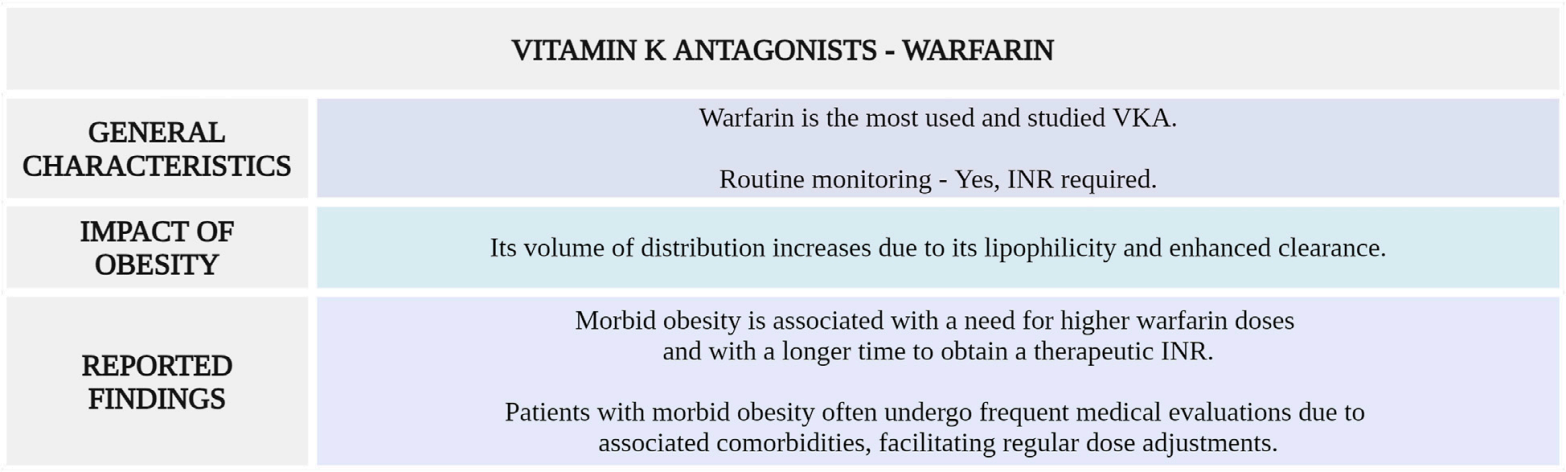
Summary of key aspects of warfarin use in morbidly obese patients with VTE. VKA, vitamin K antagonist; INR, international normalized ratio (Created with BioRender.com).

Figure 2 highlights the main considerations regarding warfarin use for VTE management in morbidly obese patients.

Although BMI can influence the required warfarin dosage, its effect on clinical practice seems limited due to regular monitoring of INR and subsequent dose adjustments to mantain therapeutic range. Additionally, given the significant comorbidities of patients with morbid obesity, they generally undergo more frequent medical check-ups, which allows for more frequent dose adjustments.

## 4 UFH use in morbidly obese patients with VTE

Intravenous UFH is used in the management of VTE, and even though it has been largely replaced by LMWH, UFH is still used for patients that are critically ill or renally impaired (George et al., 2020).

In case of acute VTE, nomogram-based dosing has been shown to improve the rapidity of achieving an anticoagulant effect and decrease the risk of VTE recurrence (Bernardi et al., 2000). Initial dosing of intravenous UFH is either weight-based or fixed. Activated partial thromboplastin time (aPTT) levels can be used for monitoring UFH therapy (Cruickshank et al., 1991; Raschke et al., 1993).

Raschke et al., in order to establish whether an intravenous dosing nomogram based on actual body weight (ABW) achieves therapeutic anticoagulation faster than a standard care nomogram, randomized patients to receive heparin in fixed doses (5,000 units bolus followed by 1,000 units/hour infusion) or adjusted doses using a weight-based nomogram (80 units/kg bolus followed by an initial infusion rate of 18 units/kg/hour). They concluded that the rate of VTE was significantly lower with the weight-adjusted heparin regimen (Raschke et al., 1993). Over time, studies have suggested that inadequate initial UFH therapy predisposes patients to late recurrence of VTE, underscoring the importance of appropriate initial dosage of heparin (Hull et al., 1997).

Although the majority of the available literature recommends the use of ABW for UFH dosing, a small proportion of the patients included in these studies were obese or morbidly obese (Riney et al., 2010). While the volume of distribution of heparin is similar to that of blood volume, accumulation of heparin in the less vascularized adipose tissue is believed to be low. Estimating the volume of distribution for heparin in obese patients becomes challenging, thus theoretically necessitating a smaller weight-based heparin dose (Shlensky et al., 2019). Consequently, consideration was given to whether dose-capping might be more appropiate for obese patients.

Available data found in literature is diverse, due to a wide variety of methodologies utilized. One study that aimed to investigate whether UFH dose-capping could be a better strategy in morbidly obese patients was published by Barletta et al. (Barletta et al., 2008). They included 101 patients, classified into morbidly obese (BMI ≥40 kg/m^2^) and non-morbidly obese (BMI <40 kg/m^2^) categories, with an indication for anticoagulation primarily consisting of the treatment of DVT or PE. By analyzing aPTT values at 6 and 12 h, they determined that both higher BMI and older age independently predict supratherapeutic aPTT levels (Barletta et al., 2008). In line with these results, Shin et al. also observed a significant difference in the mean time to achieve the first therapeutic aPTT, where patients weighing over 150 kg exhibited a longer duration compared to those in lower weight categories. However, it is important to note that this study encompassed all indications for the use of UFH (Shin and Harthan, 2015).

Another study found no significant difference in median time to achieve a therapeutic aPTT among the different BMI groups (Shlensky et al., 2019). 423 non-obese, obese, and morbidly obese patients, who received weight-based UFH using ABW without a dose-cap (80 units/kg bolus of UFH followed by a continuous infusion starting at 18 units/kg/hour) for treatment of an acute VTE, were included. Moreover, there were no differences in rates of bleeding among all three groups (Shlensky et al., 2019).

Based on the observation of a possible higher rate of supratherapeutic aPTT levels in patients with obesity, certain institutions have adopted modified weight-based dosing protocols for UFH.

For example, in Queensland, Australia, UFH doses are based on ABW and are capped depending on indication (George et al., 2020). For VTE treatment, they use capped initial bolus and maintanance doses, with 8,000 units for an 80 units/kg bolus dose and respectively, 1,500 units/h for an 18 units/kg/h infusion. In order to determine if their practices are adequate in obese patients, they performed a retrospective chart review, analysing 200 patients who were treated with UFH according to the hospital nomogram. The patients were categorized in 4 weight cohorts according to their BMI. They concluded that their nomogram was not adequate for dosing in obesity, with a need for larger absolute doses (units/h) but reduced uncapped total body weight doses (units/kg/h) in this population (George et al., 2020).

Although there are numerous studies investigating anticoagulation strategies using UFH in the obese population with VTE, the results are varied. Given the risks of excessive anticoagulation and bleeding, as well as the risk of insufficient anticoagulation, in the absence of clear recommendations emerging from literature, different attempts have been made to adopt new strategies. Thus, instead of attempting to demonstrate the necessity of capped doses in this population, some studies have modified the type of weight used for adjusting UFH doses.

For example, instead of using the ABW, some researchers investigated the utility of adjusted body weight (AjBW) (Yee and Norton, 1998; Fan et al., 2016; Alessa et al., 2021). Alessa et al. used the following equations: IBW = {45.4 + 0.89 × (height (cm) – 152.4) + 4.5 (if female)} and AjBW = {IBW +0.3 (ABW–IBW)} (Alessa et al., 2021). They studied 27 obese and 30 non-obese patients receiving treatment for acute VTE according to an internal UFH protocol, which involved an initial loading dose of 80 units/kg followed by a continuous infusion rate of 15–18 units/kg/hour. The obese patients received AjBW-based dosing of UFH while the non-obese patients received ABW-based dosing of UFH. There was no significant difference in achieving a therapeutic aPTT within the first 24 h between the two groups, suggesting that AjBW-based dosing of UFH in obese patients has comparable efficacy with ABW-based dosing of UFH in non-obese patients (Alessa et al., 2021).

In the expert position paper of the ESC Working Group on Thrombosis regarding antithrombotic therapy and body mass, there is a consensus statement that confirms the lack of validated algorithms regarding body-weight dosing of UFH in morbidly obese patients. Careful body weight estimation and frequent aPTT monitoring are recommended (Rocca et al., 2018).

UFH as a prophylactic option for VTE is administered as a subcutaneous injection, with a typycal dose of 5,000 units two or three times daily (Phung et al., 2011). The optimal dose for patients with obesity is currently unknown, with gaps in understanding whether higher doses are necessary for this population.

Lee et al. conducted a RCS on 3,056 patients admited in intensive care units and treated with standard prophylactic doses of UFH (Lee and Blanco, 2017). They were classified in two groups: 243 patients in the BMI ≥40 kg/m^2^ group and 2,813 patients in the BMI <40 kg/m^2^ group. They found no significant difference in the incidence of VTE between the groups (*p* = 0.11) (Lee and Blanco, 2017).

When trying to evaluate the safety of the use of high prophylactic dose versus standard prophylactic dose of UFH (7,500 units every 8 h versus 5,000 units every 8 h) in 320 obese patients, Regis et al. observed a signifficant difference in the incidence of bleeding between the two groups (*p* = 0.008), with no significant difference between the incidence of VTE (Regis et al., 2020). Moreover, when high-fixed prophylactic dose of UFH (7,500 units every 8 h) was compared to high-fixed prophylactic dose of enoxaparin (40 mg every 12 h) in morbidly obese patients, the incidence of major bleeding was significantly higher in the UFH group (*p* = 0.025), with no significant difference in the incidence of VTE diagnosed during hospitalization (Mason et al., 2020).

Even though high prophylactic doses of UFH are effective in the obese population, uncertainties regarding lower safety compared to standard doses warrant cautious use until larger studies are available.

**FIGURE 3:**
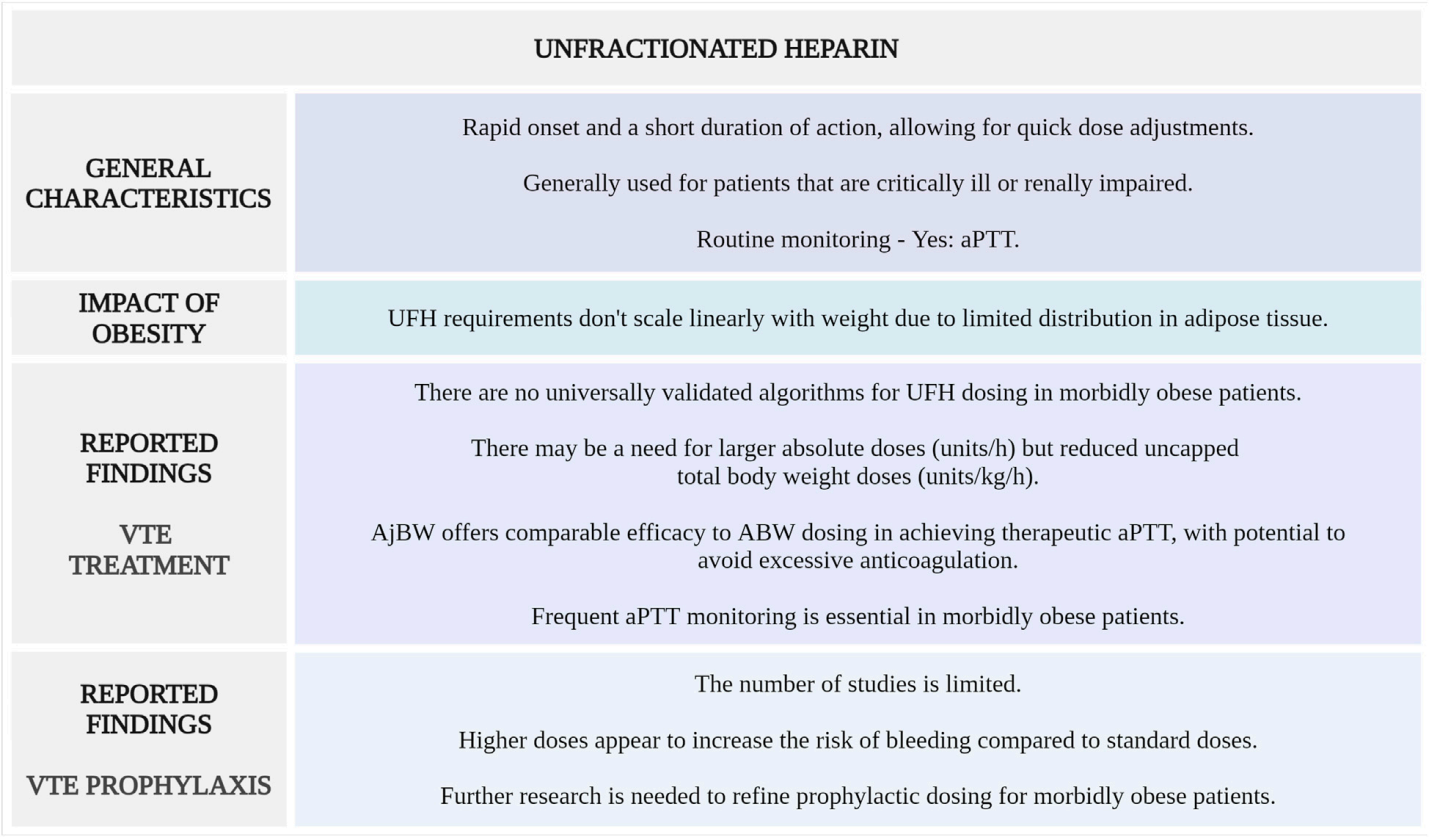
Summary of key aspects of UFH use in morbidly obese patients with VTE. aPTT, activated partial thromboplastin time; UFH, unfractionated heparin (Created with BioRender.com).

Figure 3 highlights the main considerations regarding UFH use for VTE management in morbidly obese patients.

## 5 LMWH use in morbidly obese patients with VTE

LMWH is frequently used for the prophylaxis and treatment of VTE. Individuals with a BMI exceeding 30 kg/m^2^ are often administered the same dosage as normal-weight patients (Liu et al., 2023).

Most of the available studies in the literature regarding anticoagulation with LMWH in obese patients have primarily included enoxaparin. Regarding other LMWHs, such as dalteparin, nadroparin and tinzaparin, recommendations on prophylactic or therapeutic doses in obesity are limited, and even fewer exist for the morbidly obese population.

### 5.1 Enoxaparin use in morbidly obese patients with VTE

Taking into consideration that enoxaparin is a hydrophilic molecule, its volume of distribution is roughly equivalent to plasma and does not distribute into adipose tissue (Hanni et al., 2018). This pharmacokinetic characteristic may increase the antithrombotic activity of enoxaparin in extremely obese patients compared to nonobese patients, with the risk of elevated anti-Xa levels, which are associated with increased bleeding events (Hanni et al., 2018). There is published evidence suggesting that reduced weight-based doses of therapeutic enoxaparin may be necessary in the morbidly obese category, but due to the limited evidence, reducing the therapeutic dossage of LMWH requires careful consideration. There is also published data which suggest that higher prophylactic doses of LMWH can lead to a reduced risk of VTE, without increasing the risk of bleeding (Liu et al., 2023).

In order to present the latest available data about the recommended doses of enoxaparin in morbidly obese patients, a comprehensive literature research was performed in April 2024 in PubMed database. The major search terms were as follows: “low-molecular-weight heparin”, “enoxaparin”, “venous thromboembolism”, “obese”, “obesity”, “morbidly obese”, “morbid obesity”. The above search items were connected by the logical operatos “OR” or “And”. We selected cohort studies and randomized clinical trials (RCTs) that reported enoxaparin use in adult patients with a BMI ≥40 kg/m^2^ or weight ≥120 kg for prevention or treatment of VTE. Only studies that reported anti-Xa levels as an outcome of interest were included. Studies involving patients with anticoagulation indications other than VTE were excluded.

#### 5.1.1 Enoxaparin use for VTE treatment in morbidly obese patients

There is a lack of concise recommendations regarding the management of VTE specifically in morbidly obese patients.

For example, the 2020 American Society of Hematology (ASH) guidelines for management of venous thromboembolism does not provide any remark on the obese population (Ortel et al., 2020). In their 2018 guidelines, they suggest initial LMWH dose selection according to ABW, without a capped dose, and they also suggest against using anti-Xa concentration monitoring to guide LMWH dose (Witt et al., 2018).

The American College of Chest Physicians (CHEST) does not have any recommendations regarding the use of LMWH for the obese population in either the 2016 or 2021 guidelines (Kearon et al., 2016; Stevens et al., 2021).

2019 ESC Guidelines for the diagnosis and management of acute pulmonary embolism developed in collaboration with ERS do not offer a recommendation regarding LMWH dosage in obese population (Konstantinides et al., 2019).

In the expert position paper of the ESC Working Group on Thrombosis regarding antithrombotic therapy and body mass, there has been a consensous statement that there is insufficient evidence that dose capping results in improved safety or efficacy compared with a body weight based regimen without capping in class ≥2 obesity, and that anti-Xa monitoring may be useful in class ≥3 obesity (Rocca et al., 2018).

Taking into consideration the concern for supratherapeutic plasma concentrations using the traditional dose of enoxaparin of 1 mg/kg twice daily, there have been several studies seeking to define optimal therapeutic enoxaparin dosing in the morbidly obese population. Based on our inclusion criteria, we selected 7 studies, the majority of them being small, cohort studies, with contradictory findings (Lalama et al., 2014; Lee et al., 2015; Thompson-Moore et al., 2015; Curry et al., 2018; Lee et al., 2019; Berger et al., 2021; Deal et al., 2011) (Table 1).

**TABLE 1:** Studies that reported enoxaparin use for VTE treatment in adult patients with a BMI ≥40 kg/m^2^ or weight ≥120 kg.

Reference Study design Number of patients	Inclusion BMI and/or weight	Enoxaparin dose	Patients with goal anti-Xa level	Bleeding events	Thrombotic events	Conclusion
Berger et al. (2021) RCS 102	BMI ≥40 kg/m^2^	1 mg/kg twice daily	Supratherapeutic: 42 (59.2%) Therapeutic: 25 (35.2%) Subtherapeutic: 4 (5.6%)	5	1	An initial dose of 1 mg/kg of enoxaparin might be excessive for morbidly obese patients, as numerous individuals needed a dosage adjustment following their initial anti-Xa level measurements.
Lee et al. (2019) RCS 241	BMI ≥40 kg/m^2^ Wt ≥150 kg	1 mg/kg twice daily	Supratherapeutic: 134 (55.6%) Therapeutic: 91 (37.76%) Subtherapeutic: 16 (6.64%)	10	1	Standard enoxaparin dosing in morbidly obese patients is likely to result in supratherapeutic anti-Xa levels
Curry et al. (2018) RCT 62	BMI ≥40 kg/m^2^	1 mg/kg twice daily versus 0.8 mg/kg twice daily	Dose 1 mg/kg twice daily: Therapeutic: 20 (76.9%) Dose 0.8 mg/kg twice daily: Therapeutic: 25 (89.3%)	0	0	Administering a reduced dose of enoxaparin might be a suitable dosing strategy for morbidly obese patients.
Lee et al. (2015) RCS 99	BMI ≥40 kg/m^2^ Wt ≥150 kg	CrCl ≥30 mL/min: 1 mg/kg twice daily CrCl <30 mL/min: 1 mg/kg daily	Supratherapeutic: 50 (50.5%) Therapeutic: 35 (35.4%) Subtherapeutic: 14 (14.1%)	0	NR	It is crucial to monitor anti-Xa levels to guarantee the safe and effective use of enoxaparin in obese patients. Personalizing enoxaparin dosing and assessing anti-Xa levels demand additional validation through clinical outcome research.
Thompson-Moore et al. (2015) PCS 41	BMI ≥40 kg/m^2^ Wt ≥140 kg	≥0.95 mg/kg twice daily versus <0.95 mg/kg twice daily	Dose ≥0.95 mg/kg twice daily: Supratherapeutic: 70.6% Therapeutic: 23.5% Subtherapeutic: 5.9% Dose <0.95 mg/kg twice daily: Supratherapeutic: 31.6% Therapeutic: 52.6% Subtherapeutic: 15.8%	8	0	Morbidly obese patients given the standard 1 mg/kg dose often reach supratherapeutic anti-Xa levels, increasing their bleeding risk. Starting at a lower dose and monitoring anti-Xa levels for adjustments is advisable.
Lalama et al. (2014) RCS 31	BMI ≥40 kg/m^2^ Wt ≥200 kg	CrCl ≥30 mL/min: 0.75 mg/kg twice daily CrCl <30 mL/min: 0.75 mg/kg daily	Supratherapeutic: 11 (36%) Therapeutic: 15 (48%) Subtherapeutic: 5 (16%)	0	1	Bleeding and thrombotic incidents were rare, with all patients achieving the target anti-Xa level using enoxaparin doses below 1 mg/kg.
Deal et al. (2011) RCS 26	BMI >40 kg/m^2^	Weight-based, variable doses (median 0.8 mg/kg twice daily; range 0.5–1 mg/kg twice daily)	Supratherapeutic: 38% Therapeutic: 46% Subtherapeutic: 0% Uninterpretable: 15%	6	0	Most morbidly obese patients in this group reached target or higher anti-Xa levels with doses below the recommended 1 mg/kg every 12 h. Patients experiencing bleeding events were more likely to have anti-Xa levels above the target range.

RCS, retrospective cohort study; RCT, randomized controlled trial; PCS, prospective cohort study; Wt, weight; CrCl, creatinine clearance; NR, not reported.

Only one of the selected studies was a RCT, conducted by Curry et al. (2018). They compared standard weight-based enoxaparin 1 mg/kg twice daily with a reduced dose of 0.8 mg/kg twice daily in morbidly obese patients, with a median BMI of 46.7 kg/m^2^. Both groups achieved the target anti-Xa levels in similar proportions: 89.3% and 76.9%, respectively. Dose adjustments were necessary for 9 patients, with 6 from the 1 mg/kg group, all exceeding the target levels. There were no reported cases of bleeding or thrombotic events. Based on their results, reduced dose of enoxaparin may be a reasonable dosing strategy in morbidly obese patients (Curry et al., 2018).

Berger et al. and Lee et al. conducted 2 RCSs, including 102 and 241 patients, respectively, with a BMI ≥40 kg/m^2^, in both of the studies the standard therapeutic dose of enoxaparin being used (Lee et al., 2019; Berger et al., 2021). In the study performed by Berger et al., 92.1% of patients had a BMI of ≥40–60 kg/m^2^ and 7.8% of patients had a BMI of >60 kg/m^2^. The primary endpoint of the study was the incidence of bleeding. Of the 71 patients with an initial anti-Xa level, 42 of the levels were considered supratherapeutic (59.2%). The average initial and final doses of enoxaparin were 1.0 ± 0.1 mg/kg and 0.9 ± 0.2 mg/kg, respectively. The incidence of bleeding was 4.9%, and patients who bled had higher BMIs than patients who did not bleed. Based on their results, they concluded that a standard starting dose of enoxaparin may be too high for morbidly obese patients, taking into consideration that many patients needed an adjustment to their dose after initial anti-Xa levels (Berger et al., 2021). In the study performed by Lee et al., similar conclusions were drawn. Their goal was to identify a dose of enoxaparin with the greatest chance of producing therapeutic anti-Xa levels (Lee et al., 2019). What was interesting about their study is that they divided the patients into three BMI categories. For those with a BMI of 40–50 kg/m^2^, the median therapeutic dose was 0.97 mg/kg twice daily. In subjects with a BMI of 50–60 kg/m^2^, the median therapeutic dose was 0.70 mg/kg twice daily. Finally, the median therapeutic dose for subjects with a BMI over 60 kg/m^2^ was 0.71 mg/kg twice daily. They concluded that standard dosing of enoxaparin in morbidly obese patients will most likely lead to supratherapeutic anti-Xa levels, with 53%–65% patients of all three groups having a supratherapeutic anti-Xa level before dose adjustment (Lee et al., 2019).

In a PCS made by Thompson-Moore et al., 41 patients with a BMI ≥40 kg/m^2^ or weight ≥140 kg were included (Thompson-Moore et al., 2015). The study’s main limitation was a small sample size, affecting its power to detect differences in clinical events. They recommended starting therapy at a lower dose in morbidly obese patients with VTE and monitoring anti-Xa levels to adjust enoxaparin doses accordingly (Thompson-Moore et al., 2015).

Two selected studies also included patients with renal dysfunction (Lalama et al., 2014; Lee et al., 2015). Lee et al. included 99 morbidly obese patients who received at least three doses of the standard treatment dosage of enoxaparin [creatinine clearance (CrCl) ≥30 mL/min: 1 mg/kg twice daily, CrCl <30 mL/min: 1 mg/kg daily] and had steady-state anti-Xa peak levels (Lee et al., 2015). Most of the patients (50.5%) had supratherapeutic anti-Xa levels. They concluded that monitoring anti-Xa levels is necessary to ensure the safe and effective use of enoxaparin in morbidly obese patients. Additionally, they emphasized that personalizing enoxaparin doses and monitoring anti-Xa levels should be further validated through clinical outcome studies (Lee et al., 2015). In the study performed by Lalama et al., they used their own protocol with reduced enoxaparin doses (CrCl ≥30 mL/min: 0.75 mg/kg twice daily, CrCl <30 mL/min: 0.75 mg/kg daily) for 31 patients with a weight >200 kg or BMI >40 kg/m^2^ (Lalama et al., 2014). They support using a reduced enoxaparin dose of 0.75 mg/kg in morbidly obese patients, along with anti-Xa level monitoring, but with the need of additional studies (Lalama et al., 2014).

Most of the reviewed studies support the use of a reduced therapeutic dose of enoxaparin for the management of acute VTE in morbidly obese patients. The recommendations on a specific reduced dose are not clear, with variations depending on the center where the study was conducted and on anti-Xa levels monitoring. Dosage differences across studies may affect the incidence of outcomes. The studies advocate for the importance of monitoring anti-Xa levels in this population. Bleeding events seem to be higher in patients who received the standard dose of enoxaparin but this outcome can be driven by the differences in population size between groups. Based on the available evidence, a reduced weight-based dosing for enoxaparin may seem adequate for the treatment of morbidly obese patients with VTE, but conclusions should be considered with caution until further research, especially RCTs, takes place.

#### 5.1.2 Enoxaparin use for VTE prophylaxis in morbidly obese patients

At the moment, one of the more commonly utilized anticoagulant options to prevent VTE in hospitalized and postoperative patients is a fixed, prophylactic dose of LMWH. Standard doses of enoxaparin used for VTE prophylaxis are represented by 40 mg once daily or 30 mg twice daily (Vandiver et al., 2015).

Dosing in obese or morbidly obese patients is not as clearly defined in guidelines. In the expert position paper of the ESC Working Group on Thrombosis regarding antithrombotic therapy and body mass, there is a consensus statement that obese patients are likely underdosed with fixed once-daily LMWH regimens (Rocca et al., 2018). They agreed that higher fixed daily or body weight adjusted dosing regimens have proven to be efficacious in high-risk, moderate- and morbidly-obese patients, suggesting an empirically increase in standard prophylaxis dose by 30%. They also agreed that for morbidly obese patients, anti-Xa measurement can provide therapeutic guidance, even though the therapeutic anti-Xa range and sample timing in severely obese patients remains unknown (Rocca et al., 2018).

Based on our inclusion criteria, we selected 5 studies, with only two of them including medical inpatients (Simone et al., 2008; Freeman et al., 2012; Steib A et al., 2016; Gelikas et al., 2017; Gibson et al., 2021) (Table 2).

**TABLE 2:** Studies that reported enoxaparin use for VTE prophylaxis in adult patients with a BMI ≥40 kg/m^2^ or weight ≥120 kg.

Reference Study design Number and type of patients	Inclusion BMI and/or weight	Enoxaparin dose	Patients with goal anti-Xa level	Bleeding events	Thrombotic events	Conclusion
Gibson et al. (2021) PCS 80, medical inpatients	BMI ≥40 kg/m^2^	40 mg twice daily versus 0.5 mg/kg daily	Dose 40 mg twice daily: Supratherapeutic: 10% Therapeutic: 72.50% Subtherapeutic: 20% Dose 0.5 mg/kg daily: Supratherapeutic: 2.5% Therapeutic: 70% Subtherapeutic: 25%	0	0	Both weight-based and twice-daily escalated dosing of enoxaparin seem effective in reaching target anti-Xa levels in hospitalized patients, with no safety issues observed. Further research is necessary to assess the clinical implications of these findings.
Gelikas et al. (2017) PCS 54, bariatric	BMI ≥40 kg/m^2^	40 mg daily versus 60 mg daily	Dose 40 mg daily: Therapeutic: 80.6% Dose 60 mg daily: Therapeutic: 91.3%	1	0	Given the lack of adequate data on the clinical effectiveness and safety of various dosing regimens, both studied regimens are viable options for preventing VTE after bariatric surgery.
Steib et al. (2016) RCT 135, bariatric	BMI >40 kg/m^2^	4000 IU daily versus 6000 IU daily versus 4000 IU twice daily	Dose 4000 IU daily: Therapeutic: 12.8% Dose 6000 IU daily: Therapeutic: 56.4% Dose 4000 IU twice daily: Therapeutic: 27.3%	1	0	Administering a daily single dose of 6000 IU of enoxaparin enabled the majority of patients to achieve the target range of anti-Xa activity without raising the risk of bleeding.
Freeman et al. (2012) PCS 31, medical inpatients	BMI ≥40 kg/m^2^	40 mg daily versus 0.4 mg/kg daily or 0.5 mg/kg daily	Dose 40 mg daily: Subtherapeutic: 82% Dose 0.4 mg/kg daily: Subtherapeutic: 36% Dose 0.5 mg/kg daily: Subtherapeutic: 13%	0	0	In severely obese, critically ill patients, enoxaparin administered at 0.5 mg/kg once daily is more effective than both fixed-dose and lower-dose regimens in achieving the desired anti-Xa levels.
Simone et al. (2008) PCS 40, bariatric	BMI >40 kg/m^2^	40 mg twice daily versus 60 mg twice daily	Dose 40 mg twice daily: Therapeutic: 45% Dose 60 mg twice daily: Therapeutic: 73%	1	NR	Enoxaparin at a dose of 60 mg every 12 h outperformed the 40 mg dosage in achieving therapeutic anti-Xa levels and preventing subtherapeutic concentrations. Nevertheless, the 60 mg group experienced some instances of supratherapeutic levels. Further research involving larger cohorts of morbidly obese patients is necessary to explore the correlation between anti-Xa concentrations and clinical outcomes.

RCS, retrospective cohort study; RCT, randomized controlled trial; PCS, prospective cohort study; IU, international unit; NR, not reported.

In a prospective, multi-center trial study, Gibson et al. included 80 patients with a BMI ≥40 kg/m^2^ (Gibson et al., 2021). In the study, 40 patients were given 40 mg of enoxaparin twice daily, while another 40 received a weight-based dose of 0.5 mg/kg. This was the first study to compare these escalated enoxaparin doses. They found no significant difference in the percentage of patients reaching the target anti-Xa levels (72.5% vs 70.0%, *p* = 0.72), and no bleeding or thrombotic events were reported. The researchers recommend further studies to evaluate if either regimen offers clinically meaningful benefits (Gibson et al., 2021).

The other study which only included medical inpatients with a BMI ≥40 kg/m^2^ was published by Freeman et al. (2012). The study prospectively compared three enoxaparin dosing regimens to achieve target peak anti-Xa levels. Patients were assigned to receive either a fixed dose of 40 mg daily, a weight-based lower dose of 0.4 mg/kg daily, or a weight-based higher dose of 0.5 mg/kg daily. There were no bleeding or thrombotic events reported. The results indicated that a daily dose of 0.5 mg/kg enoxaparin in patients with an average BMI of over 60 kg/m^2^ was superior in achieving target peak anti-Xa levels compared to both fixed dosing and the lower weight-based dose (Freeman et al., 2012). However, their study is limited by the small number of patients and the lack of clinical outcomes allowing correlation with anti-Xa levels.

In our search, we identified three studies evaluating enoxaparin as thromboprophylaxis in bariatric patients which only included individuals with a BMI ≥40 kg/m^2^ (Simone et al., 2008; Steib et al., 2016; Gelikas et al., 2017). All dosing regimens were different between studies, and different conclusions were drawn. We found only one RCT, performed by Steib et al., who compared enoxaparin 4000 UI once daily, 4000 UI twice daily, and 6000 UI once daily in 135 morbidly obese patients undergoing bariatric surgery, with a significantly large proportion of patients reaching therapeutic anti-Xa levels with enoxaparin 6000 UI once daily. Only a few bleeding events were observed in any group during a follow-up period of 30 days (Steib et al., 2016).

Gelikas et al., in a PCS investigating two doses regimens, 40 mg daily and 60 mg daily, respectively, consider both doses to be reasonable choices for venous thromboembolic events prophylaxis after bariatric surgery (Gelikas et al., 2017).

Comparatively to Steib et al. and Gelikas et al., Simone et al. conducted a study following the outcomes of using enoxaparin 40 mg twice daily and 60 mg twice daily (Simone et al., 2008). Even though the 60 mg twice daily dose has proven superior to 40 mg twice daily dose in achieving therapeutic levels on anti-Xa, they suggest that additional studies are needed, considering the supratherapeutic levels of anti-Xa obtained with the 60 mg twice daily dose (Simone et al., 2008).

Overall, conclusions mainly relied on biochemical measures of anti-Xa levels, given the few bleeding and thrombotic events reported. In this matter, some precautions should be taken, considering that recommended anti-Xa levels for prophylactic usage are poorly defined in literature. In the selected studies, the same ranges of anti-Xa levels were used for both once or daily enoxaparin administrations, and this could also be a limitation. Generally, prophylactic doses of enoxaparin should be increased for patients with obesity. However, given the small number of studies that have included only patients with morbid obesity, along with their limitations, well-designed RCTs are necessary to obtain clearer dose recommendations.

**FIGURE 4:**
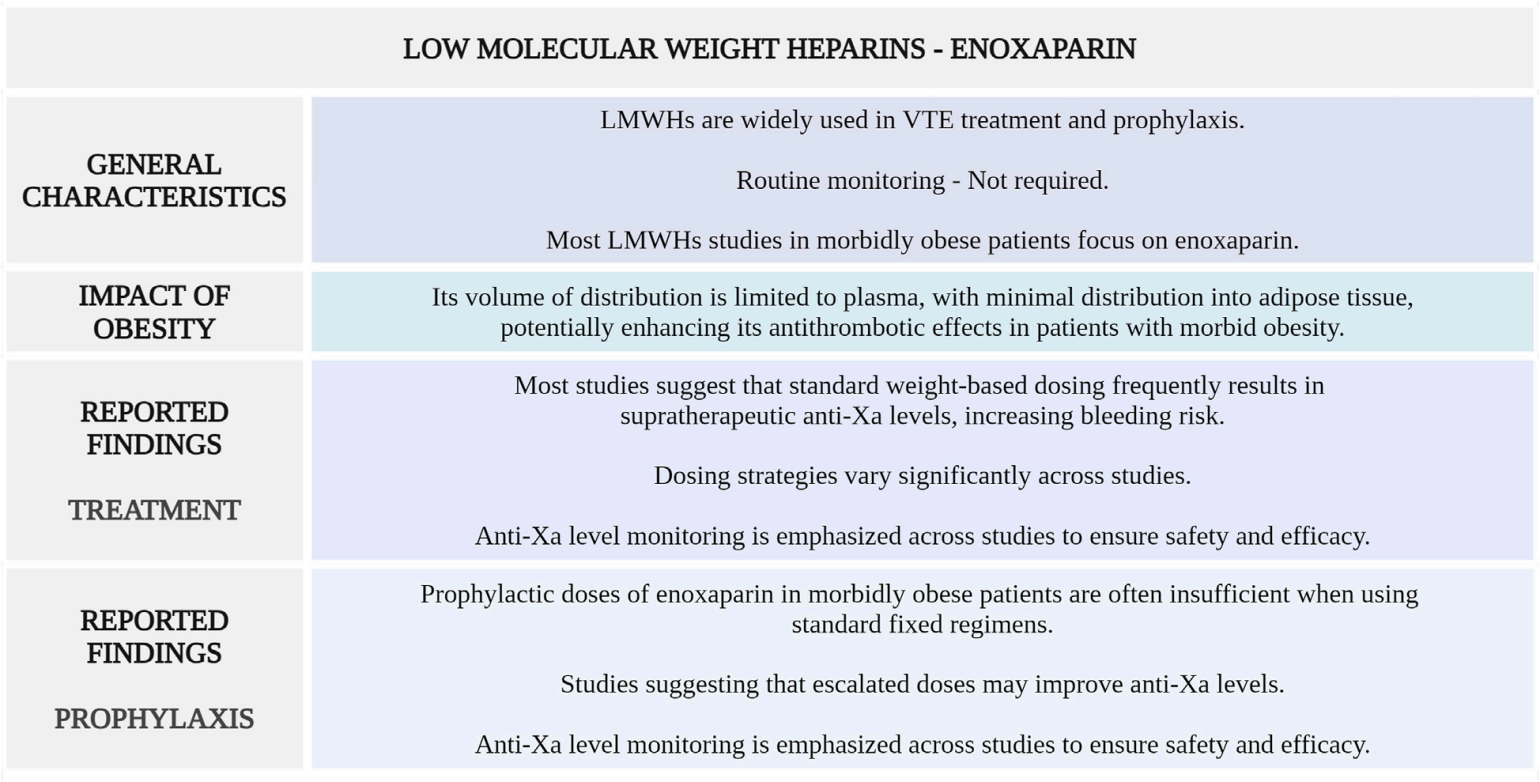
Summary of key aspects of enoxaparin use in morbidly obese patients with VTE. LMWHs, low-molecular-weight heparins; VTE, venous thromboembolism; anti-Xa, anti-factor Xa (Created with BioRender.com).

Figure 4 highlights the main considerations regarding enoxaparin use for VTE management in morbidly obese patients.

## 6 Fondaparinux use in morbidly obese patients with VTE

Fondaparinux is a synthetic factor Xa inhibitor indicated for various clinical conditions, including the prevention and treatment of VTE (Nadar et al., 2009). Its mechanism of action involves enhancing antithrombin III activity to selectively inhibit factor Xa, thereby suppressing thrombin generation without directly affecting thrombin activity (Zhang et al., 2019).

It has predictable pharmacokinetics, with rapid and complete absorption after subcutaneous administration, and typically does not require routine monitoring. Its renal clearance necessitates dose adjustments in patients with impaired kidney function (Bauersachs, 2023). Fondaparinux offers several advantages, including a minimal risk of heparin-induced thrombocytopenia (HIT), stable pharmacokinetics enabling once-daily dosing, and fewer drug-drug interactions compared to other anticoagulants (Zhang et al., 2019). While bleeding may occur, its incidence is influenced by the timing of administration, particularly in postoperative settings. Unlike heparin, fondaparinux’s effects cannot be neutralized by protamine (Bauersachs, 2023; Ghaziri et al., 2023).

The pharmacokinetics of fondaparinux are influenced by body weight, as its distribution is limited to blood volume and it is predominantly excreted through the kidneys. In patients with higher body weight, the drug’s clearance increases proportionally, requiring adjustments in dosing to maintain therapeutic plasma levels (Nadar et al., 2009).

Patients weighing between 50 and 100 kg typically exhibit normal pharmacokinetics, with a standard daily dose of 7.5 mg being appropriate for the treatment of VTE. In those weighing over 100 kg, increased clearance necessitates a higher dose of 10 mg daily to achieve effective anticoagulation. These weight-based dose adjustments ensure the drug maintains its efficacy while minimizing the risk of adverse effects, provided renal function is normal (Rocca et al., 2018; Yin et al., 2024).

Although there is evidence supporting the efficacy of increasing the dose of fondaparinux in patients weighing over 100 kg, the data includes a limited number of patients with a weight exceeding 120 kg or a BMI over 40.

One study performed by Davidson et al. compared fondaparinux with enoxaparin and UFH for treating VTE in obese and non-obese patients, analyzing outcomes for recurrence and major bleeding by weight (≤100 kg, >100 kg) and BMI (<30, ≥30 kg/m^2^) (Davidson et al., 2007). Although the study showed similar rates of recurrence and bleeding across weight and BMI groups, suggesting that standard fondaparinux doses are as effective and safe as heparins for VTE in obese and non-obese patients, the small number of patients with a BMI >50 limits the applicability of these findings and may miss subtle differences in safety or efficacy (Davidson et al., 2007).

In patients with morbid obesity and VTE, until further studies confirm the safety and efficacy of fondaparinux in this population, it would be more cautious to opt for an alternative anticoagulant, such as LMWH.

The same precaution applies to the prophylactic dose of 2.5 mg, which may not be sufficient for patients with extreme obesity. A retrospective study evaluated anti-factor Xa levels in morbidly obese patients (BMI ≥40 kg/m^2^) receiving the standard prophylactic dose of fondaparinux (2.5 mg daily) for VTE prevention. Among 47 values analyzed, 47% were below, 43% within, and 11% above the target range, with no thromboembolic events reported during hospitalization. The findings suggest that the standard dose may be suboptimal in morbidly obese patients, highlighting the need for further research into weight-based dosing adjustments (Martinez et al., 2011).

## 7 DOACs use in morbidly obese patients with VTE

DOACs have been approved for the prophylaxis and treatment of VTE, becoming the preferred antithrombotic treatment option over the VKAs, mostly due to their ease of use, efficacy and safety profiles (Talerico et al., 2024). Additionally, DOACs are increasingly utilized as an alternative for primary prevention of VTE, in patients undergoing hip and knee arthroplasty, data suggesting at least comparable efficacy and no heightened risk of bleeding when compared to LMWH (Highcock et al., 2020). DOACs can also be used as an extended anticoagulation option for secondary prevention of VTE (Mai et al., 2019).

The benefits associated with DOACs include fixed dosing regimens, a wider therapeutic window without the need for regular monitoring, and fewer drug-drug and drug-food interactions than VKA (Zhao et al., 2023).

However, there is a lack of data on the clinical pharmacokinetics, pharmacodynamics, efficacy, and safety of DOACs in patients with obesity, and in particular, those with severe obesity (Rosovsky et al., 2023). Taking into consideration that DOACs are lipophilic drugs, concerns have been raised regarding an increase in their volume of distribution and the risk of undercoagulation in obese population (Gouju and Legeay, 2023).

Over time, there have been discrepancies in recommendations regarding the utilization of DOACs in patients with obesity. In 2016, the International Society on Thrombosis and Haemostasis (ISTH) suggested that DOACs should not be used in patients with a BMI of >40 kg/m^2^ or a weight of >120 kg, given the limited data available at that time (Martin et al., 2016). They also recommended checking a drug-specific peak and trough level if DOACs had to be used in this population, with the suggestion to switch to a VKA rather than adjusting the dose of DOAC if the drug-specific level was found to be below the expected range (Martin et al., 2016). The 2021 guidance statement of ISTH for use of DOACs in patients with a BMI of >40 kg/m^2^ or a weight of >120 kg suggested that standard doses of apixaban or rivaroxaban should be used for VTE treatment and prevention, without regularly following the peak or trough drug-specific DOAC levels. They also advised against using dabigatran, edoxaban, or betrixaban for VTE treatment and prevention in this population (Martin et al., 2021).

The ISTH 2021 guidelines were based on a literature review conducted up to 1 August 2020 (Martin et al., 2021). Since then, new studies have emerged that have investigated the efficacy and safety of DOACs in the management of VTE in patients with morbid obesity, with particular focus on apixaban and rivaroxaban.

In order to highlight the latest available data about the recommended doses of DOACs in morbidly obese patients, a literature research was performed in April 2024 in PubMed database. The major search terms were as follows: “direct oral anticoagulant”, “DOAC”, “apixaban”, “rivaroxaban”, “dabigatran”, “edoxaban”, “venous thromboembolism”, “obese”, “obesity”, “morbidly obese”, “morbid obesity”. The above search items were connected by the logical operatos “OR” or “And”. We selected cohort studies and RCTs that reported DOACs use in adult patients with a BMI ≥40 kg/m^2^ or weight ≥120 kg for treatment or secondary prevention of VTE. We excluded studies that did not report outcomes regarding efficacy or safety, did not include subgroup analyses specifically on patients with a BMI ≥40 kg/m^2^ or weight ≥120 kg, or included patients with atrial fibrillation and did not perform a specific analysis focusing solely on patients with venous thromboembolism (Table 3).

**TABLE 3:** Studies on the efficacy and safety of DOACs in patients with VTE with a weight ≥120 kg or BMI ≥40 kg/m^2^.

Reference Study design	Patients	Intervention	Conclusions
Sperry et al. (2024) RCS	Acute VTE patients stratified in two BMI groups: -low BMI group (BMI <40 kg/m^2^) -high BMI group (BMI ≥40 kg/m^2^)	Apixaban, rivaroxaban and dabigatran in the low BMI group. Apixaban and rivaroxaban in the high BMI group.	No notable disparity in VTE recurrence or major bleeding associated with BMI was observed in patients treated with DOACs. This study suggests that DOACs could be a safe and effective treatment for VTE in individuals with obesity.
Martin et al. (2023) RCS	5626 VTE patients with a Wt ≥120 kg or BMI ≥35 kg/m^2^ (2,321 patients with a BMI ≥40 kg/m^2^)	Apixaban (n = 472), rivaroxaban (n = 1,343), dabigatran (n = 52), edoxaban (n = 1) versus warfarin (n = 3,758)	Patients with obesity treated for VTE using DOACs and warfarin showed similar rates of recurrent VTE: for BMI ≥40 kg/m^2^, 4.4% versus 3.5% respectively (*p* = 0.34) In those with BMI 35 to <40 kg/m^2^, DOACs were associated with significantly less bleeding than warfarin (*p* < 0.001), while similar rates of major bleeding occurred between the two groups in other BMI categories.
Crouch et al. (2021) RCS	1099 VTE patients with a Wt ≥120 kg or BMI ≥40 kg/m^2^	Apixaban (n = 314) versus warfarin (n = 785)	Apixaban demonstrated a significantly prolonged time to recurrent VTE (*p* = 0.018) and was linked to a decreased risk of recurrent VTE compared to warfarin (*p* = 0.04). There were no notable distinctions in major bleeding, clinically relevant non-major bleeding, or all-cause mortality.
Weaver et al. (2022) RCS	1,281 acute VTE patients with a Wt >120 kg or BMI >40 kg/m^2^	Rivaroxaban (n = 487) versus warfarin (n = 785)	There was no significant difference observed in morbidly obese patients treated with either rivaroxaban or warfarin for VTE regarding the hazard of VTE recurrence or major bleeding. Both agents may be considered appropriate treatment options in this population.
Scott et al. (2022) RCS	247 acute VTE patients with BMI ≥40 kg/m^2^	DOAC (n = 129) versus VKA (n = 118)	There was no statistically significant difference in the risk of VTE events (*p* = 0.07) between the use of DOACs and warfarin.
Berger et al. (2022) RCS	6,058 patients with BMI ≥40 kg/m^2^	Rivaroxaban (n = 3,565) versus warfarin (n = 2,493)	Patients with morbid obesity and VTE who started treatment with rivaroxaban experienced a reduced risk of VTE recurrence and similar rates of major bleeding compared to those started on warfarin, over a 12-month follow-up period.
Anusim et al. (2022) RCS	499 acute VTE patients with BMI >40 kg/m^2^	Rivaroxaban (n = 296) or apixaban (n = 203)	No association between rivaroxaban and apixaban with an increase in VTE recurrence. No statistically significant differences in bleeding rates or mortality between the rivaroxaban and apixaban groups.
Cohen et al. (2021) Post hoc analysis of the AMPLIFY trial	VTE patients with a Wt ≥120 kg or a BMI >40 kg/m^2^	Apixaban (n = 144) versus warfarin/enoxaparin (n = 146)	Among morbidly obese patients, apixaban demonstrated similar rates of recurrent VTE or VTE-related death and significantly lower rates of major bleeding or clinically relevant non-major bleeding. These findings align with the primary outcomes observed in the AMPLIFY trial.
Costa et al. (2020) Cohort analysis	13,510 obese VTE patients of whom 3,394 had a BMI ≥40 kg/m^2^	Rivaroxaban (n = 1,697) versus warfarin (n = 1,697)	Rivaroxaban showed a decreased risk of VTE recurrence at 3, 6, and 12 months in morbidly obese patients, with no significant difference in major bleeding events during these time intervals.
Perino et al. (2021) RCS	6,934 acute VTE patients with a Wt ≥120 kg (≥120 kg - <140 kg, n = 4,767; ≥140 kg, n = 2,167)	≥120 kg - <140 kg: DOAC (n = 1938) versus warfarin (n = 2,829); ≥140 kg: DOAC (n = 725) versus warfarin (n = 1,442)	In patients weighing between 120 kg and less than 140 kg, DOAC treatment was linked to lower rates of major bleeding (*p* = 0.0217) and clinically relevant non-major bleeding (*p* = 0.0022), along with fewer recurrent VTE events numerically. For patients weighing 140 kg or more, there were no significant differences in outcome rates, although there were numerically fewer cases of major bleeding.
Samaranayake et al. (2021) Matched case–control study	150 acute VTE (PE) patients with a Wt >120 kg or a BMI >40 kg/m^2^	DOAC (n = 104) versus warfarin (n = 46)	The incidence of recurrent VTE and bleeding events at 6 months did not differ significantly between DOACs and warfarin.
Spyropoulos et al. (2019) RCS	2,890 matched pairs of morbidly obese (Wt >120 kg or a BMI >40 kg/m^2^) VTE patients initiating rivaroxaban or warfarin	Rivaroxaban (n = 2,890) versus warfarin (n = 2,890)	Morbidly obese patients who started or continued treatment with either rivaroxaban or warfarin experienced comparable risks of recurrent VTE and major bleeding. With fewer than 1% of rivaroxaban-treated patients undergoing anti-Xa testing, clinicians can be confident that those with morbid obesity and VTE on rivaroxaban have outcomes similar to standard warfarin, without needing routine anti-Xa monitoring. Rivaroxaban associated with fewer hospitalizations and fewer outpatient visits compared to warfarin.
Kushnir et al. (2019) RCS	366 VTE patients with a BMI ≥40 kg/m^2^	Apixaban (n = 47), rivaroxaban (n = 152) versus warfarin (n = 167)	Similar incidence of VTE (*p* = 0.74) and major bleeding (*p* = 0.77 between the treatment cohorts.
Sa et al. (2019) RCS	133 acute VTE patients with a Wt >120 kg	Rivaroxaban, apixaban, edoxaban versus warfarin (n = 62)	Similar risks of recurrent VTE (*p* = 0.337). and of major bleeding (*p* = 0.337) between the treatment cohorts.

RCS, retrospective cohort study; Wt, weight.

In line with the ISTH 2021 recommendations, a study published in 2024 by Sperry et al. concluded that apixaban and rivaroxaban are effective and safe treatment options for acute VTE patients with a BMI ≥40 kg/m^2^ (Sperry et al., 2024). The study included 165 acute VTE patients with a BMI ≥40 kg/m^2^ and 320 acute VTE patients with a BMI <40 kg/m^2^, with no difference in rates of VTE recurrence or major bleeding between the groups. Only one of the patients was treated with dabigatran. No difference was found in VTE recurrence or major bleeding between patients receiving apixaban compared to rivaroxaban (Sperry et al., 2024). Similar to their results, apixaban and rivaroxaban were found safe and effective in morbidly obese population in a RCS performed by Anusim et al. (2022). They included 499 acute VTE patients with BMI >40 kg/m^2^ treated with apixaban or rivaroxaban, followed at 60 days for VTE ocurrence or bleeding events. They found no statistically significant differences in VTE recurrence, bleeding rates or mortality, between the rivaroxaban and apixaban groups (Anusim et al., 2022).

The other studies found in literature compared DOCSs to warfarin, having similar results. DOACs, especially rivaroxaban and apixaban, were associated either with a similar risk of VTE recurrence or bleeding, or with a lower risk (Kushnir et al., 2019; Sa et al., 2019; Spyropoulos et al., 2019; Costa et al., 2020; Cohen et al., 2021; Crouch et al., 2021; Perino et al., 2021; Samaranayake et al., 2021; Berger et al., 2022; Scott et al., 2022; Weaver et al., 2022; Martin et al., 2023).

In a RCS conducted by Crouch et al., 1099 morbidly obese patients treated with apixaban or warfarin for acute VTE were included. Apixaban use was associated with a lower risk of VTE recurrence, with no significant differences in bleeding risk or all-cause mortality between groups (Crouch et al., 2021).

The Apixaban for the Initial Management of Pulmonary Embolism and Deep-Vein Thrombosis as First-Line Therapy (AMPLIFY) trial is a well known double-blind, randomized, multicenter study that compared efficacy and safety of apixaban with those of conventional therapy in 5,395 patients with acute VTE. They concluded that apixaban was noninferior to conventional therapy for the treatment of acute VTE and was associated with significantly less bleeding (Agnelli et al., 2013). Cohen et al. performed a *post hoc* analysis of the AMPLIFY trial in order to explore the efficacy, safety and exposure of apixaban for the treatment of VTE in patients with a body weight ≥120 kg or BMI >40 kg/m^2^. The findings were consistent with the main results of the AMPLIFY trial, supporting the use of apixaban in morbidly obese patients with acute VTE (Cohen et al., 2021).

A case report published in 2023 indicated that DOACs can achieve therapeutic anti-Xa levels even in patients with a BMI greater than 70 kg/m^2^ (Kingsley et al., 2023). The report described a case involving a 243 kg woman with a history of menorrhagia, who was admitted with acute intermediate-high risk PE and lower extremity DVT. She was effectively treated with rivaroxaban, maintaining therapeutic anti-Xa levels during the acute treatment phase. However, due to the onset of heavy menstrual bleeding, her treatment was adjusted to a maintenance dose of 20 mg daily earlier than planned. The patient had no further complications and showed no signs or symptoms of recurrent VTE (Kingsley et al., 2023).

Regarding primary prevention of VTE, where the use of DOACs is limited to elective hip and knee arthroplasty, the ISTH 2021 guidelines suggest that apixaban and rivaroxaban administered in standard doses are appropiate anticoagulant options regardless of high BMI and weight (Martin et al., 2021).

**FIGURE 5:**
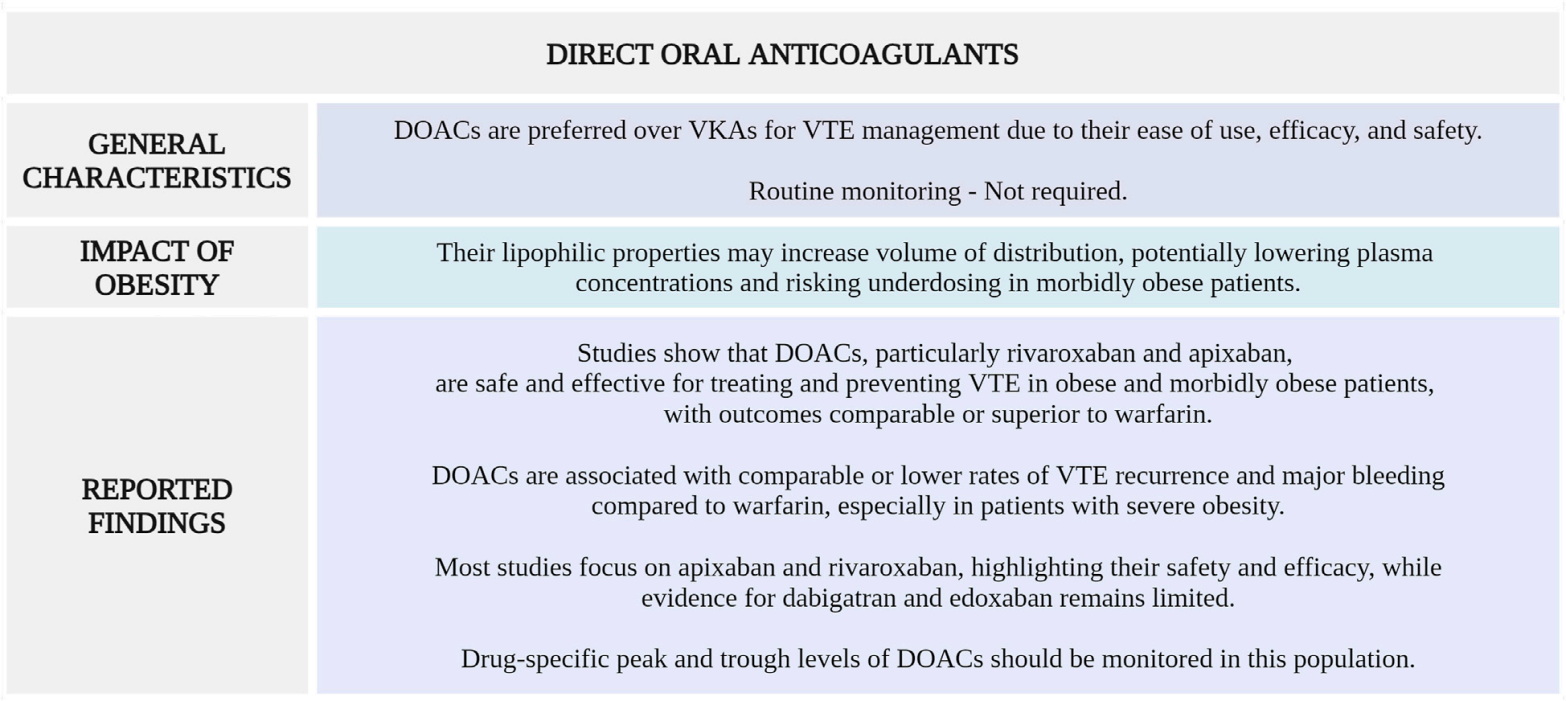
Summary of key aspects of DOACs use in morbidly obese patients with VTE. DOACs, direct oral anticoagulants; VKAs, vitamin K antagonists; VTE, venous thromboembolism (Created with BioRender.com).

Figure 5 highlights the main considerations regarding DOACs use for VTE management in morbidly obese patients.

## 8 Anticoagulation in special morbidly obese patients populations with VTE

### 8.1 Anticoagulation in morbidly obese patients with cancer

VTE stands as a leading cause of mortality among cancer patients, despite being a largely preventable disease (Guntupalli et al., 2023). The thrombotic risk varies according to the cancer type, administered cancer therapies, and patient-related risk factors, including comorbidities such as obesity (Abdol Razak et al., 2018). A crucial point to consider is that cancer patients not only face an elevated risk of thrombosis but also a higher risk of bleeding complications, making anticoagulation management a complex issue (Angelini et al., 2019).

In recent years, numerous guidelines and consensus papers shared recommendations regarding the management of VTE in cancer patients, emphasising the need of an individualized treatment (Carrier et al., 2021; Lyman et al., 2021; Falanga et al., 2023; Key et al., 2023).

Although there are clear guidelines regarding the type of anticoagulant recommended based on various clinical scenarios and patient risks, recommendations for patients with obesity are limited, especially for those with morbid obesity.

The 2021 updated Canadian Expert Consensus on the treatment algorithm in cancer-associated thrombosis recommend to consider LMWH in cancer patients with a weight >150 kg (Carrier et al., 2021).

In 2023, the European Society for Medical Oncology (ESMO) published a clinical practice guideline regarding VTE management in cancer patients, also including special populations such as obese patients (Falanga et al., 2023). In cancer patients with a weight >120 kg or BMI ≥40 kg/m^2^, they recommend the use of LMWH calculated based on the ABW, without capping at a maximum dose. They also suggest considering the use of DOACs in this population, but with caution (Falanga et al., 2023).

### 8.2 Anticoagulation in morbidly obese patients with renal impairment

Obesity is a risk factor for both the onset and progression of chronic kidney disease (CKD), directly through obesity-related glomerulopathy and indirectly through complications associated with obesity, such as atherosclerosis, hypertension, and type 2 diabetes (Nawaz et al., 2022).

Renal impairment can result in increased exposure to anticoagulants due to reduced renal clearance, and current clinical evidence remains unclear on the benefits and drawback of various treatment strategies (Ma et al., 2024). This issue is further compounded by a significant lack of data concerning the obese and morbidly obese populations.

Accurately determining the glomerular filtration rate (GFR) in obese patients is essential. Ideally, GFR should be directly measured using substances that are freely filtered by the kidneys without being secreted or resorbed. However, these compounds are typically used only in research settings (Wuerzner et al., 2011). In clinical settings, GFR is typically estimated using endogenous markers such as SCr through various formulas (Levey et al., 2019). However, estimating GFR in obese individuals presents challenges due to factors affecting SCr production, including extreme body size and undetected relative sarcopenia. For this reason, SCr levels can be unreliable for estimating GFR. Moreover, the standard formulas used in clinical practice are not specifically calibrated for the obese population (Fernández et al., 2023).

The most used formulas are represented by Modification of the Diet in Renal Disease (MDRD) formula, the Chronic Kidney Disease Epidemiology Collaboration formula (CKD-EPI) and the Cockcroft-Gault (CG) formula.

In contrast to the CG formula, the MDRD or CKD-EPI formulas do not necessitate body weight data for estimating GFR (Florkowski and Chew-Harris, 2011). In a study of 54 morbidly obese patients who had the CrCl measured by 24-h urine collection, the CG equation was found to overestimate the CrCl. However, integrating lean body weight into the CG equation yielded an unbiased and precise value (Demirovic et al., 2009).

MDRD and CKD-EPI estimate GFR using an unique body surface area (BSA) of 1.73 m^2^ that represent an average surface area of 25-year-old Americans from more than 100 years ago (Korhonen et al., 2023). This value is significantly smaller than the average BSA of today’s individuals and it can lead to underestimation of GFR (López-Martínez et al., 2019).

To enhance the applicability of these formulas for obese individuals, some studies suggest modifying them by using lean weight rather than total weight for the CG formula, and by de-indexing MDRD and CKD-EPI formulas (Musso and González-Torres, 2019; Domislovic et al., 2022). Further research is needed to determine whether indexed GFR is superior to unindexed GFR in determining important clinical outcomes. Additionally, when considering the use of DOACs, it is essential to note that the pivotal trials relied on the CG formula for estimating GFR.

### 8.3 Anticoagulation in bariatric surgery patients

As global obesity rates have been consistently rising in recent years, there has been an increase in bariatric surgeries, which are currently considered the most effective treatment for achieving lasting weight loss and alleviating related health conditions (Leven et al., 2020). Most bariatric procedures are performed laparoscopically, increasing intraabdominal pressure and the risk of thrombosis, thus making bariatric surgery a high-risk procedure for thromboembolic events (Ruiz-Tovar and Llavero, 2017).

The anatomical alterations resulting from bariatric procedures have various physiological impacts on drug absorption and subsequent bioavailability. These effects are contingent upon both the physicochemical characteristics of the drug and the properties of the gastrointestinal tract (Martin et al., 2017).

Considering the limited data available regarding the pharmacokinetic and pharmacodynamic changes of oral anticoagulants in this scenario, prophylactic or therapeutic anticoagulation in bariatric patients becomes challenging. The 2021 ISTH guidance statement suggests initial parenteral anticoagulation in the early postoperative period, with a potential transition to VKA or DOACs after a minimum of 4 weeks of parenteral therapy. Due to absorption concerns, they recommend monitoring DOAC levels (Martin et al., 2021). Building upon these recommendations and incorporating data obtained through a systematic review, Leong R et al. provided additional insights into the postoperative management of thromboembolic risk (Leong et al., 2022). For patients who have had highly malabsorptive or complex surgical procedures, it is recommended to use parenteral anticoagulation with LMWH or fondaparinux during the early postoperative period (Leong et al., 2022). In cases of acute VTE, they suggest administering a therapeutic dose of LMWH without capping the dose up to 140 kg, or using a weight-adjusted dose of fondaparinux during the first 1–4 weeks after diagnosis. Following this initial parenteral anticoagulation period, they recommend starting a DOAC (apixaban, rivaroxaban, or edoxaban) and monitoring peak drug levels 3–5 days after beginning treatment. If the drug levels are within the expected range, a second peak level check should be done 4–6 months post-surgery. If two or more DOACs show low peak levels, switching to warfarin should be considered (Leong et al., 2022).

Some recent studies investigated the use of DOACs as a prohyplactic anticoagulant medication following bariatric surgery. In a retrospective analysis of data from 5,017 patients who had undergone bariatric surgery, Surve et al. investigated the safety and efficacy of 2.5 mg apixaban twice daily for a total of 30 days starting on day 3 postoperatively (Surve et al., 2022). They concluded that this strategy appears to be safe and effective. However, they were aware that their study had some limitations, such as its retrospective nature, not taking into consideration patient-related and procedure-related predisposing risk factors, and assessing only symptomatic thromboembolic events without using ultrasound or CT scans (Surve et al., 2022). The BARIVA (Bariatric Rivaroxaban) study is the first trial that investigated the efficacy and safety of a 10 mg daily rivaroxaban dose in a randomized setting in patients after bariatric surgery (Kröll et al., 2023). Patients were randomly allocated in a 1:1 ratio to receive either 7 days (short prophylaxis) or 28 days (long prophylaxis) of treatment. They concluded that once-daily VTE prophylaxis with 10 mg of rivaroxaban was effective for both short and long prophylaxis groups. A strength of their research is the use of bilateral compression ultrasonography screening to detect asymptomatic thrombotic events. However, a limitation is the absence of an LMWH treatment group (Kröll et al., 2023).

**FIGURE 6:**
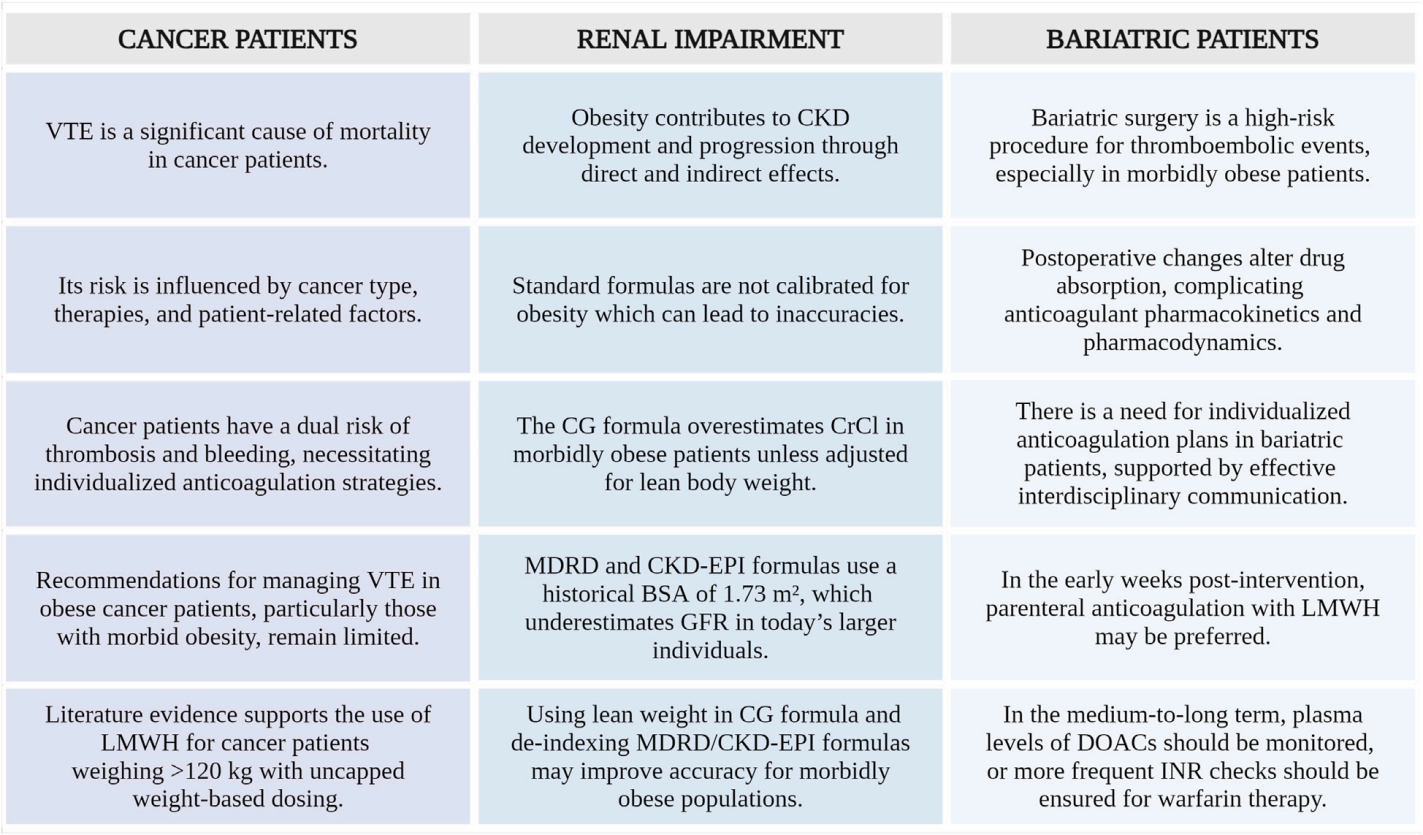
Summary of key aspects in special morbidly obese patients populations with VTE. VTE, venous thromboembolism; CKD, chronic kidney disease; CG, Cockcroft-Gault; CrCl, creatinine clearance; MDRD, Modification of the Diet in Renal Disease; CKD-EPI, Chronic Kidney Disease Epidemiology Collaboration; BSA, body surface area; GFR, glomerular filtration rate; LMWH, low-molecular-weight heparin; DOACs, direct oral anticoagulants; INR, international normalized ratio (Created with BioRender.com).

Figure 6 outlines the essential considerations for VTE management in specific subgroups of morbidly obese patients.

## 9 Recommendations

Based on the limited available data, which predominantly consists of retrospective cohort studies with significant heterogeneity in the doses used, and with the reservation imposed by the necessity of further RCTs, we consider the following anticoagulation strategies to be suitable for patients with a weight ≥120 kg or a BMI ≥40 kg/m^2^ and VTE:

• Warfarin: frequent INR monitoring is mandatory, taking into consideration that morbid obesity is associated with a need for higher warfarin doses and with a longer time to obtain a therapeutic INR.
• UFH as thromboprophylaxis: standard doses of UFH should be used until further research, taking into consideration the uncertainties regarding lower safety of high prophylactic doses.
• UFH as treatment: there may be a need for larger absolute doses (units/h) but reduced uncapped total body weight doses (units/kg/h) in this population. AjBW rather than ABW can be used to calculate UFH dosing in order to avoid excessive anticoagulation. Frequent aPTT monitoring is required.
• Enoxaparin as thromboprophylaxis: higher fixed daily prophylactic doses of 60–80 mg (administered once daily or divided into two doses) may be used.
• Enoxaparin as treatment: reduced weight-based enoxaparin dose of 0.8 mg/kg twice daily, without a dose cap, can be administered. Anti-Xa levels monitoring may be useful in this special population.
• Cancer patients: it is advisable to administer a weight-based therapeutic dose of enoxaparin 1 mg/kg twice daily, without a dose cap.
• Fondaparinux: in patients with morbid obesity, fondaparinux should be avoided for the prophylaxis or treatment of VTE until further studies confirm its safety and efficacy in this population. LMWH can be used as an alternative.
• DOACs as treatment: standard doses of apixaban or rivaroxaban can be used for VTE treatment. Monitoring of peak and/or trough anti-Xa activity is recommended. If concentrations are too low, switching to VKA may be necessary. The data on dabigatran and edoxaban are scarce.
• Patients with renal impairment: dosing should be adapted according to the recommendations specific for each type of anticoagulant. Using lean weight rather than total weight for the CG formula and de-indexing MDRD and CKD-EPI formulas may be suitable for an accurate estimation of GFR.

**FIGURE 7:**
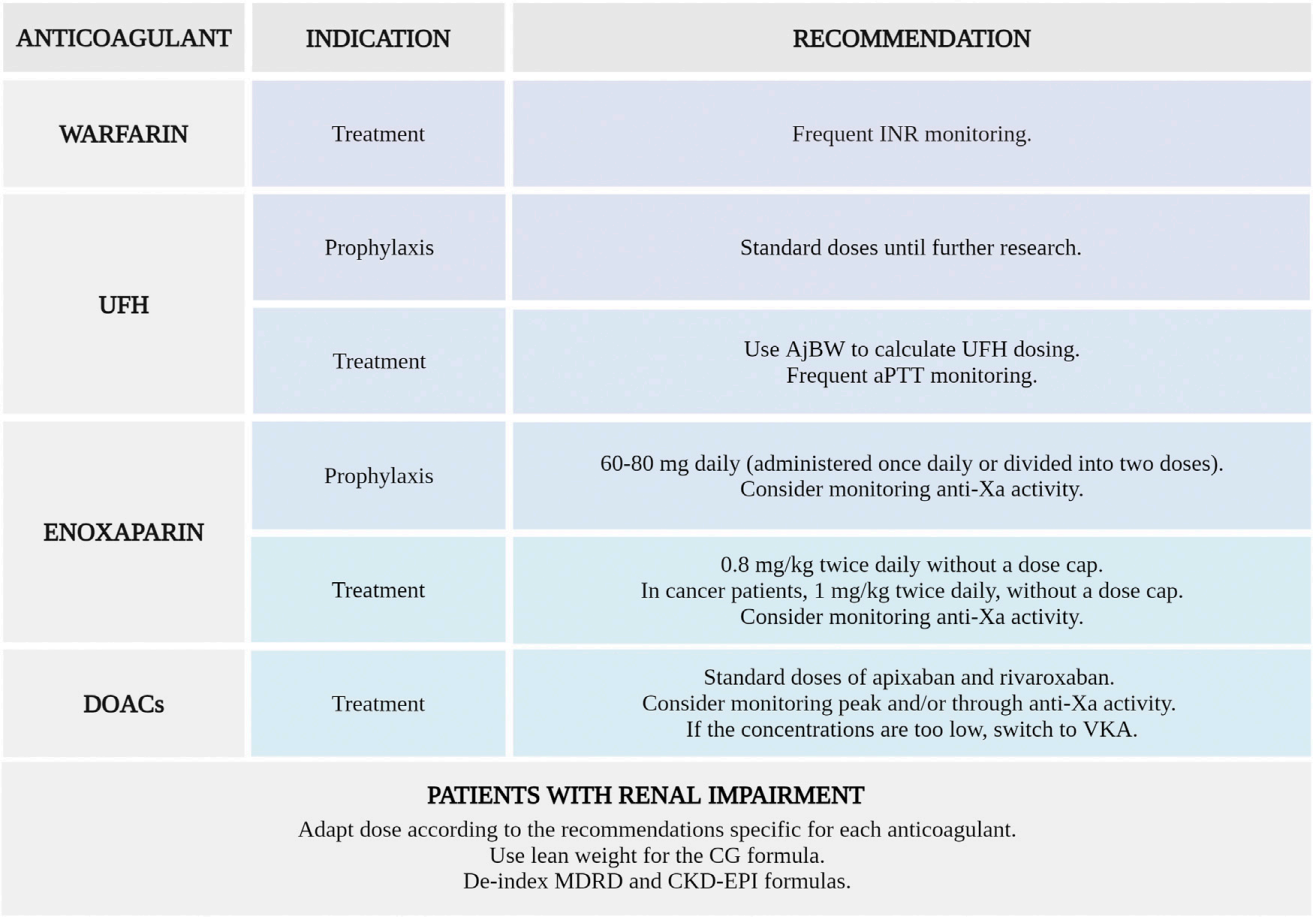
Anticoagulation strategies suitable for the prophylaxis or treatment of venous thromboembolism in patients with a weight ≥120 kg or a BMI ≥40 kg/m^2^. INR, international normalized ratio; UFH, unfractionated heparin; AjBW, adjusted body weight; aPTT, activated partial thromboplastin time; anti-Xa, anti-factor Xa; DOACs, direct oral anticoagulants; VKA, vitamin K antagonists; CG, Cockcroft-Gault; MDRD, Modification of the Diet in Renal Disease; CKD-EPI, Chronic Kidney Disease Epidemiology Collaboration (Created with BioRender.com).

These recommendations are summarized in Figure 7.

## 10 Conclusion

Obesity is rapidly increasing worldwide, being a well known risk factor for new and recurrent VTE. In summary, this review highlights the intricate relationship between obesity and VTE, shedding light on the challenges regarding the anticoagulation management. It is evident that current recommendations for anticoagulant treatment in patients with morbid obesity and VTE are still unclear, with a need for an individualized approach and careful monitoring in this special population in order to ensure an optimal balance between the benefits and risks of anticoagulant therapy.

## Glossary

ABW: actual body weight
AMPLIFY: Apixaban for the Initial Management of Pulmonary Embolism and Deep-Vein Thrombosis as First-Line Therapy
ASH: American Society of Hematology
AjBW: adjusted body weight
BARIVA: Bariatric Rivaroxaban
BMI: body mass index
BSA: body surface area
CG: Cockcroft-Gault
CHEST: American College of Chest Physicians
CKD: chronic kidney disease
CKD-EPI: Chronic Kidney Disease Epidemiology Collaboration
CRP: C-reactive protein
CrCl: creatinine clearance
DOACs: direct oral anticoagulants
DVT: deep vein thrombosis
ERS: European Respiratory Society
ESC: European Society of Cardiology
ESMO: European Society for Medical Oncology
GFR: glomerular filtration rate
IBW: ideal body weight
IL-6: interleukin-6
INR: international normalized ratio
ISTH: International Society on Thrombosis and Haemostasis
LMWH: low-molecular-weight heparin
MDRD: Modification of the Diet in Renal Disease
NSAIDs: nonsteroidal anti-inflammatory drugs
PAI-1: plasminogen activator inhibitor-1
PCS: prospective cohort study
PE: pulmonary embolism
PTS: post-thrombotic syndrome
RCS: retrospective cohort study
RCT: randomized controlled trial
SCr: serum creatinine
TF: tissue factor
TNF-alpha: tumor necrosis factor alpha
UFH: unfractionated heparin
VKAs: vitamin K antagonists
VTE: venous thromboembolism
WHO: world health organization
aPTT: activated partial thromboplastin time
anti-Xa: anti-factor Xa
